# Tact Training with Augmentative Gestural Support for Language Disorder and Challenging Behaviors: A Case Study in an Italian Community-Based Setting

**DOI:** 10.3390/jcm13226790

**Published:** 2024-11-11

**Authors:** Laura Turriziani, Rosa Vartellini, Maria Grazia Barcello, Marcella Di Cara, Francesca Cucinotta

**Affiliations:** 1 Anmic Riabilitazione, San Giovanni in Fiore, 87055 Calabria, Italy; turrizianilaura@gmail.com (L.T.); r.vartellini@gmail.com (R.V.); mariagraziabarcello@gmail.com (M.G.B.); 2 IRCCS Neurolesi Bonino Pulejo, S.S. 113 Via Palermo, C. da Casazza, 98124 Messina, Italy; francesca.cucinotta@irccsme.it

**Keywords:** augmentative and alternative communication, tact training, rehabilitation, language disorder

## Abstract

Background: Gestures or manual signing are valid options for augmentative and alternative communication. However, the data in the literature are limited to a few neurodevelopmental disorders, and less is known about its application in the community setting. Objectives: This case report explores the feasibility and preliminary efficacy of tact training with augmentative gestural support intervention for a child affected by a language disorder with challenging behaviors in a community setting. Methods: Baseline assessments were conducted using the Verbal Behavior Milestone Assessment and Placement Program (VB-MAPP) and Griffiths Mental Developmental Scale-III (GMDS-III). The patient received six months of standard treatment, consisting of neuropsychomotor and speech therapy each twice a week, with improved cooperation in proposed activities, but no improvement in language. Afterward, a total of 24 sessions of tact training with augmentative gestural support interventions were performed. Data were collected by two independent observers and analyzed to measure language and behavioral outcomes. Results: VB-MAPP scores increased form minimal communication and social interaction at T0 (baseline) to improved compliance but unchanged language skills at T1 (after standard therapy). After tack training with augmentative gestural support (T2), VB-MAPP scores showed significant improvements, with notable increases in verbal operants, independence in communication, and intersubjectivity skills. GMDS-III scores at T2 also demonstrated growth in social, communicative, and cognitive skills. Additionally, challenging behaviors were reduced by more than 70% and nearly resolved by the end of the intervention. Conclusions: Personalized approaches appear to be essential for interventions tailored to developmental age. Further research is needed to determine the effectiveness of these approaches for other neurodevelopmental disorders, identify patient characteristics that may be predictors of outcomes to tailor the intervention, and explore the generalization of the results obtained with these strategies.

## 1. Introduction

Communication deficits are among the main causes of challenging behavior in children with neurodevelopmental disorders [1,2]. Difficulty with communication often leads children to express their needs in alternative ways, sometimes learning to achieve their goals through problematic behaviors [3]. These behaviors can be exacerbated by a lack of adequate support, particularly within the legal system [4]. Effective communication interventions are therefore essential, not only to facilitate social interactions but also to reduce the risk of misunderstandings or adverse outcomes in complex settings [4,5].

This study aims to investigate the effectiveness of augmentative gestural support in enhancing communication skills and reducing challenging behaviors in children with language disorders.

Augmentative and alternative communication (AAC) strategies, such as gestures, have shown significant potential in addressing communication deficits that often lead to problematic behaviors in various neurodevelopmental conditions. From a behavioral perspective, communication is considered a behavior that can be enhanced by manipulating the environment and modifying antecedents and consequences [6,7,8].

Timely intervention can also improve neuropsychological development and ameliorate outcomes [9]. According to Skinner, verbal behavior (VB) includes not only vocal language but also gestures and symbols [10]. AAC systems can support communication in individuals with neurodevelopmental disorders, reducing dysfunctional behaviors and improving coping skills [11,12]. The literature recommends early AAC interventions to prevent developmental delays and mitigate problematic behaviors [13,14,15].

Reinforcing communicative interactions leads to new learning opportunities [16,17], even in the presence of neurological impairments [18,19,20]. Learned communication skills must be generalized across various life contexts [21], and using gestures and images can promote greater independence and access to higher verbal functions [22,23]. Progress in verbal skills facilitates new behavioral and cognitive acquisitions, including forming internal representations and recognizing others’ mental states [24,25,26].

AAC strategies are crucial for enhancing and ensuring social participation despite neuropsychiatric impairment [12,27]. AAC strategies include high-tech options, such as voice synthesizers and specialized software, alongside low-tech methods, such as picture exchange systems and gestures. While gestures are often effective, they are primarily used in cases of profound hearing loss as a permanent alternative to spoken language. In neurodevelopmental disorders, however, they are typically applied as a temporary method aimed at fostering vocal language development rather than replacing it.

Recent studies confirm the importance of gestures in communication improvement [28,29,30,31,32,33], as a gestural communication often precedes verbal production in children, promoting linguistic and intellectual development [30,34]. Specifically, gestures have proven effective in increasing verbal and vocal behaviors in individuals with autism and other developmental disabilities, enhancing communication levels, and reducing problematic behaviors [35,36,37,38].

Finally, neuroscientific studies indicate that visuo-gestural communication [39,40,41] engages the same brain areas as vocal language, thereby strengthen neural connections for communication [30,42,43]. Visual cues such as gestures also increase joint attention, early communication, and self-control, proving to be a valuable AAC tool [44,45]. However, most research on gestural communication has been conducted in controlled settings, with a growing need for studies evaluating the effectiveness in real-world settings, such as community-based services. This type of research is crucial to understanding how to better tailor therapies to local resources and the specific needs of the assisted population. Augmentative gestural support has shown efficacy in addressing broader language deficits, supporting children with various language impairments to acquire functional communication skills and reduce maladaptive behavior [46]. Studies demonstrate that AAC interventions are effective in community-based settings as they can be adapted to the specific needs and resources of each population [47]. This flexibility makes AAC strategies accessible in everyday settings, fostering sustainable progress in communication and behavioral. However, most research on specific language disorder (SLD) interventions has been conducted in controlled settings, highlighting a growing need for evaluations in community-based contexts to better align therapies with real-world demands.

The present case report aimed to investigate the efficacy of augmentative gestural support within a community-based service setting to improve word learning and communication skills in a child with SLD and global developmental delay (GDD), while also assessing its impact on reducing challenging behaviors.

## 2. Case Description

This single-case study was conducted with C., a five-year-old Italian child who received a diagnosis of SLD and GDD with a hyperkinetic behavioral pattern. This diagnosis was established at 48 months from local public health services. Observations and assessments in our setting further characterized his developmental profile and communicative behaviors, confirming the presence of significant language and behavioral challenges.

C.’s early developmental history included a normotensive pregnancy and full-term birth, followed by early surgical intervention for vesicoureteral reflux. His behavior was marked by high activity levels, emotional dysregulation, and abrupt topic shifts, often triggered by communication challenges.

C. presented a hyperkinetic behavioral pattern with a rapid shift of activities, reduced tolerance to denials and frustrations, and abrupt shifts from one topic to another. Additionally, he had not yet achieved sphincter control.

Expressive communication skills were limited to single words and simple gestures. To request help, he would lead an adult to the desired object, while refusal involved pushing items away or shouting. Notably, there was a clear discrepancy between his expressive and receptive abilities. While his expressive language was minimal, his verbal comprehension appeared adequate, allowing him to understand both simple and complex commands. He demonstrated the ability to perform basic cognitive tasks, including serializations, associations, and categorizations within major lexical categories. Baseline testing with the Verbal Behavior Milestone Assessment and Placement Program (VB-MAPP) [48] further highlighted his communicative profile, showing very low levels of spontaneous manding; he often acted independently to fulfill his needs, seldom seeking assistance from others. His play was primarily solitary, and he exhibited minimal social interactions, with gestures serving as his primary form of communication support. The assessment categorized him as a “passive conversationalist”, characterized by responsive rather than assertive communicative acts, emphasizing his tendency to react to others’ initiations rather than starting interactions on his own.

Written informed consent was obtained from the child’s parents.

## 3. Method

### 3.1. Assessment

Two independent observers (L.T. and RV.) collected all clinical data during the unstructured observations and assessments using the VB-Mapp [48] and Griffiths Mental Developmental Scale-III (GMDS-III) [49] conducted over 10 sessions. The VB-Mapp assesses language and social skills, while the GMDS-III provides a comprehensive assessment of developmental levels in children from 0 to 6 years of age. During the VB-MAPP pre-test phase, the child’s abilities were assessed across the following milestones: (a) Mand, (b) Tact, (c) Echoic, (d) Listener, (e) Independent Play, (f) Socialization, (g) Social Play, (h) Visual Perception, (i) Imitation, and (l) Vocal Behavior. Data were recorded through direct observation of the child.

Due to C.’s limited cooperation with structured tasks, it was not possible to conduct a formal evaluation of his communicative and linguistic skills. Consequently, several structured tests were excluded, as they required sustained participation and verbal responses, which C. was unable to provide. These tests included the following:

- Fanzago Articulation Test: This test is designed to compile a phonetic inventory by having the child name images representing words with various phonemes. This test relies on verbal naming and was thus excluded [50].
- Phono-Lexical Test (TFL): This test is aimed at assessing language comprehension and production. This test was also excluded due to the child’s reluctance to engage in structured responses [51].
- Rustioni Linguistic Comprehension Test, TCGB (Grammar Comprehension Test for Children), and Peabody Picture Vocabulary Test: These tests evaluate morphosyntactic and receptive language abilities but require structured, verbal responses, which the child was not able to provide [52].
- Assessment of Social-Communicative Skills (ASCB Questionnaire), First Language Test (TPL) [53], and First Vocabulary Inventory Questionnaire (PVB): These questionnaires assess early vocabulary and socio-conversational skills; however, they were excluded as the child’s age exceeded the normative limits of these instruments.

Considering these limitations, the GMDS-III was selected to obtain a global assessment of the child’s developmental functioning, as this scale allows for observation-based scoring across multiple developmental domains. The GMDS-III was deemed appropriate due to its flexibility in capturing developmental milestones without requiring high levels of verbal cooperation, aligning with the child’s specific profile and ability to cooperate during assessments.

**Figure 1:**
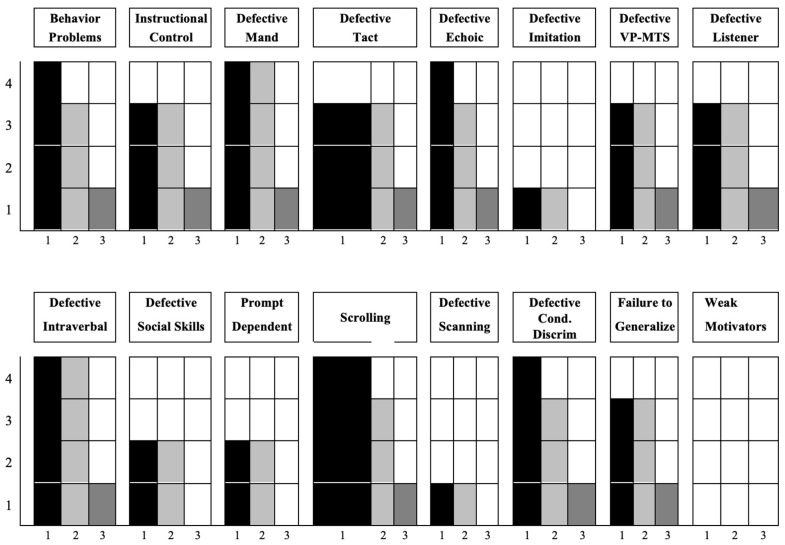

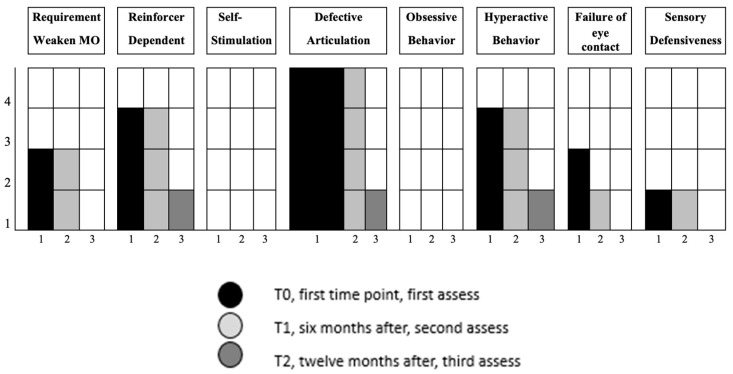
Barriers assessment. VB-MAPP at baseline (T0), after six months (T1), and after twelve months (T2). The barriers assessment determines the presence of 24 learning and language acquisition barriers frequently faced by children with Autism or developmental delays.

At baseline, the barriers assessment (Figure 1) identified 24 learning and language acquisition barriers faced by the child.

**Figure 2:**
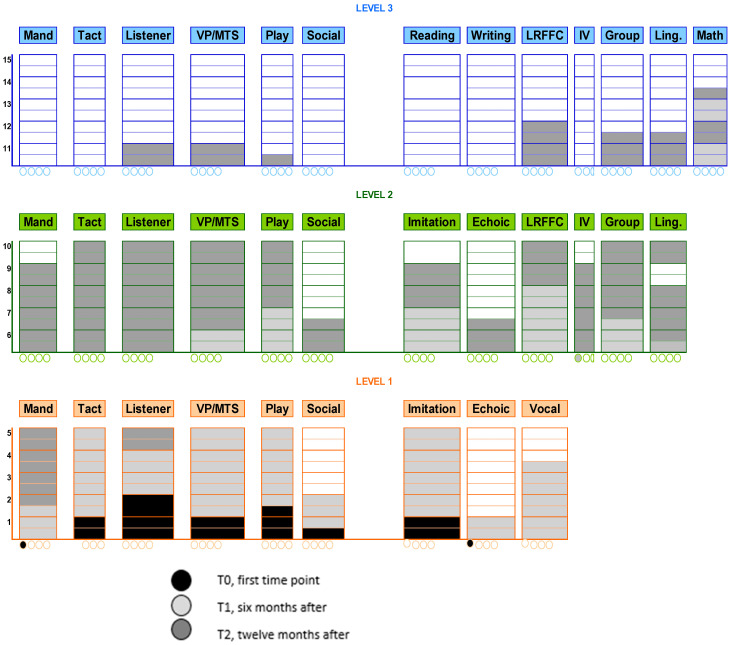
VB-MAPP at T0-T1-T2. This figure illustrates the progression of the child’s performance based on the VB-MAPP assessment over three key time points.

VB-MAPP results at baseline (T0), after six months (T1), and twelve months’ post-intervention (T2) were reported in Figure 2 and Table 1 and Table 2.

Each milestone was score based on specific criteria. The scores range from 0 to 1, with 0 indicating an absence of or incorrect response, 0.5 for a partial response, and 1 for an accurate and independent response.

The master scoring form, depicted as a bar graph, reflected the child’s overall profile, with each assessment highlighted in a different color.

**Figure 3:**
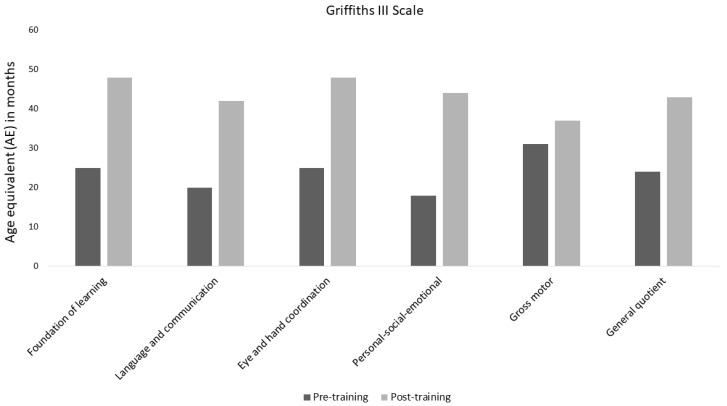
Griffiths Mental Developmental Scale-III at T1 and T2. In the GMDS-III assessment, the child obtains an equivalent age (AE) in months for each skill. In the bar graph, the AE values for each subscale obtained at six months and after twelve months have been placed side by side.

At T0, it was not possible to complete the GMDS-III developmental assessment due to limited collaboration and prolonged challenging behaviors, such as refusing proposed activities, expressed through complaining, rejecting materials, and consistently avoiding the task. Following T0, the child participated in t treatment as usual (TAU) within the Italian community service center, receiving neuro-psychomotor training and speech therapy, each with two 45-min sessions per week. After six months (T1), repeated unstructured clinical observation by the same two independent observers indicated improved behavioral compliance but no improvement in language skills on the VB-MAPP. At this point, both VB-MAPP and GMDS-III assessments were successfully completed, and the results are shown in Table 3 and Figure 3.

### 3.2. Intervention

Based on the child’s behavioral features, and verbal and nonverbal communication profile, the setting and treatment approach were adjusted to address specific needs. The treatment-as-usual (TAU) intervention was structured following applied behavior analysis (ABA) principles and implemented over six months, with four weekly sessions lasting 45 min each. This frequency was maintained throughout the intervention period. The sessions followed a structured format: (a) a pairing phase, during which the therapist created a positive association with the environment, toys, and themselves as a preferred stimulus to enhance the child’s engagement; (b) a tact training phase, in which images of frequently used bisyllabic words were introduced to develop foundational communication skills; (c) a free play period following the training, designed to encourage spontaneous use of newly acquired skills.

In the experimental phase, target bisyllabic minimal words and accompanying gestures were selected based on C.’s daily communication needs, specific interests, and motor abilities. The selection of linguistic targets was inspired by the child’s primary needs and natural context, identifying high-frequency bisyllabic words that correspond to items easily found in the child’s daily environment. Lexical labels were tailored to C.’s oral and vocal imitation capabilities, assessed through the Orofacial and Oroverbal Praxis Test [54], where C. was asked to reproduce orofacial motor sequences. The most accurate movements were noted, and linguistic targets were chosen to match the articulation point of the correctly imitated movements. Additionally, gestural labels were developed to correspond to each syllable, aligning gestures with the mode of articulation to represent voiced/voiceless traits and consonant features (e.g., stops, fricatives, affricates, nasals, vibrants, or approximants). This selection process aimed to ensure that each word was both functional and accessible for the child, focusing on words that could be represented with clear, manageable gestures. Gestures were chosen to match C.’s emerging motor skills, enhancing his ability to imitate and produce them consistently. For instance, bisyllabic words such as ‘fish’ were paired with gestures that involved simple hand movements within the child’s capability, minimizing frustration and promoting successful imitative responses. Additionally, syllables were prioritized over phonemes as they represent a more natural unit of articulation, making them more recognizable and easier to reproduce for C., who demonstrated greater success with syllable repetition. This approach not only facilitated motor learning but also aimed to build confidence in vocal production, encouraging C. to experiment with and expand his vocal repertoire. The child was then encouraged to imitate the signs while attempting an approximate vocal response. Correct responses, defined as accurate reproduction of syllabic signs, were recorded with a (+) mark, while incorrect responses were marked with a (-). Correct responses were reinforced with a preferred tangible item, while incorrect responses were addressed using physical prompts, guiding the child to replicate the correct sign.

Additionally, spontaneous vocal productions without signing were differentially reinforced, providing both the requested item and social reinforcement. A continuous reinforcement schedule was implemented: each learn unit [55] was followed by social reinforcement.

The mastery criterion for each training target was set at 90% accuracy for two consecutive sessions of 10 learning units (LUs), after which prompt fading was applied. Independent correct responses, defined as spontaneous gesture selection and tact production, were recorded with a (+), and new signs representing additional words were introduced upon reaching a 100% accuracy in a 10 LU session. This incremental approach aimed to build a more extensive lexical repertoire. After reaching the mastery criterion for the training targets, a post-test was conducted using the same procedures as the pre-test to evaluate retention and generalization of skills.

In addition, data on the duration of challenging behaviors were systematically collected and recorded throughout the treatment. The interobserver agreement (IOA) was calculated using the data collected by two independent observers by dividing the number of agreements by the total number of agreed data added to the disagreed data and multiplying by 100 [56]. In cases where disagreements arose between the two observers during data collection, the recorded session footage was reviewed collaboratively, and a consensus was reached through discussion. This process ensured that all observations were accurately coded according to predefined criteria. IOA was calculated based on the final agreed-upon data, yielding an overall IOA score of 95%. Data on the child’s responses, including correct and incorrect productions of syllables and gestures, were collected manually by two independent observers using a standardized observation checklist. The checklist was designed to systematically document each learning unit, marking each response as correct (+) or incorrect (-) based on predefined accuracy criteria. For consistency and accuracy in data collection, all sessions were video-recorded, allowing observers to review and confirm responses post-session. Additionally, IOA was calculated to ensure reliability, using a standardized formula where agreements were divided by the sum of agreements and disagreements, multiplied by 100, yielding an IOA score of 95%.

Behavioral data, including the duration and frequency of challenging behaviors, were recorded systematically throughout the intervention period. Observers employed a time-sampling method, using a timer and frequency counter app to capture the duration of specific behaviors. To ensure consistency, each observer followed a coding protocol for behavior measurement.

### 3.3. Results

In Figure 2, the VB-MAPP scores at T0, T1, and T2 illustrate the progression of C.’s linguistic, communicative, and other milestone abilities.

At T0, C. did not emit independent mands but required physical prompts to request a visible item controlled by the operator. He could label two familiar people without echoic prompts (e.g., “mum” and “dad”) and responded by turning toward the operator when called by name across five trials (listener response). C. tracked moving stimuli visually for two seconds in five instances over a 30-min observation (visual perception/matching-to-sample). Within the play milestone, he engaged in solitary play with five different objects in a 30-min session. Socially, he showed interest in the movements of people around him at least twice but fewer than five times over the same observation period. He was able to imitate gross motor movements modeled by an adult, but no echoic production was observed. In terms of spontaneous vocal behavior, C. produced five distinct vocal sounds, averaging about 10 sounds per hour. In summary, at baseline, C.’s abilities were limited, particularly in independent communication and social engagement. His interactions relied on prompts, and his social interest was minimal. These findings indicate that, initially, C. lacked the foundational verbal and social skills necessary for independent communication, aligning with his “passive conversationalist” profile.

At T1, C. produced three mands for visible reinforcements with a vocal prompt (e.g., “What do you want?”). He could spontaneously label (tact) 10 common objects and perform four different motor actions upon verbal instruction (listener response). He successfully matched 25 identical images in a field of six (visual perception/matching-to-sample) and played independently with five objects based on their function. Socially, he indicated a desire for physical play twice in one hour and imitated 10 different motor actions with objects in a set of three when prompted (“do this”). Emerging echoic skills were observed, with syllables and letter sounds, and he spontaneously produced word approximations. In the listener responding by function, feature, and class (LRFFC) milestone, he selected the correct item from a set of five for 25 different statements. He began following basic group rules with a vocal prompt (“put the objects away” and “line up”). Family members reported understanding his tacts for 10 named but non-present objects. By T1, C. demonstrated significant growth in communication skills, moving from dependence on prompts to a more autonomous use of mands and tacts. His ability to follow verbal instructions and engage in play reflects progress in both motor and cognitive skills, suggesting that he was beginning to develop foundational skills that support verbal imitation and functional communication. Socially, his interest in group activities and basic rule-following signals increased engagement and adaptability in social settings.

At T2, C. spontaneously emitted 15 different mands under various motivating operations within a 30-min period. He labeled approximately 200 objects, images, and actions, and, upon verbal instruction, he could choose items based on shape and color from a set of six for four different colors and shapes. He spontaneously recreated activities and manipulation tasks demonstrated by adults or peers twice and engaged in pretend play on two occasions. Socially, he began initiating mands with peers and imitated functional skills in natural environments. In echoic behavior, he accurately repeated bisyllabic words. Additionally, he correctly selected 25 items in response to listener demands based on function, feature, and category and answered 25 different “what” questions. In group settings, he was able to respond to two different group questions or instructions within a group of three children. C. also demonstrated mastery in using past and present tense in spontaneous speech. By T2, C. showed substantial improvements in independent communication and social skills. His increase in spontaneous mands and labels reflects a shift toward functional communication, and his ability to initiate interactions indicates greater social competence. The development of echoic and symbolic play skills suggests improved motor planning and cognitive flexibility, foundational skills for more advanced social and communication behaviors. His increased verbal output and grammatical mastery align with intervention goals, demonstrating the efficacy of the ABA and AAC strategies used to enhance language and social engagement in children with SLD and GDD.

**Figure 4:**
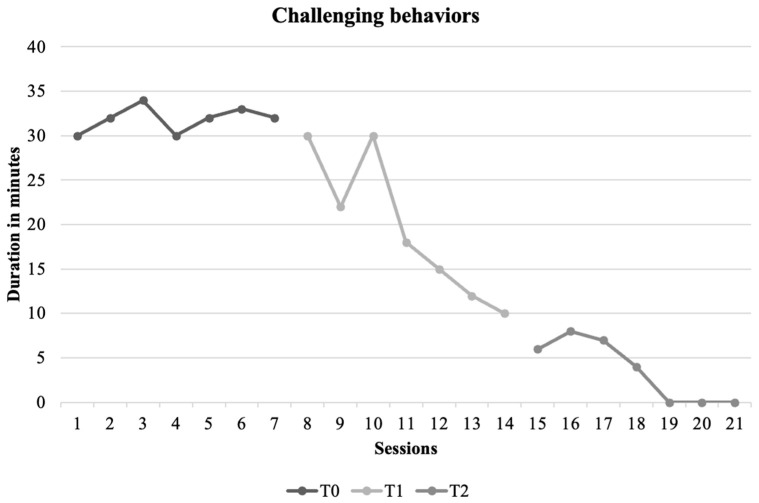
Challenging behaviors measured at baseline (T0), after six months (T1) and after twelve months (T2). The duration was measured as they were persistent, and it was not possible to measure their frequency.

These results indicate a steady progression in C.’s communication skills and social interactions as well as a reduction in challenging behaviors across the assessment points. At T0, his abilities were limited. However, by T2, C. demonstrated significant improvements in both independent manding and functional play, as well as an emerging ability to communicate effectively in social contexts (Figure 4). These changes align with our intervention goals, supporting the efficacy of combining ABA and AAC strategies to enhance functional communication and social engagement in children with SLD and GDD.

## 4. Discussion

### 4.1. Integration of ABA and AAC in Enhancing Communication and Reducing Problem Behaviors

This case study focused on C., a five-year-old Italian child diagnosed with SLD and GDD at 48 months of age. Over six months, C. underwent an intervention based on ABA combined with AAC strategies, specifically using marked syllables and signs, chosen to support his emerging motor imitation skills. Numerous studies support the integration of ABA with AAC for improving communication outcomes in children with intellectual and developmental disabilities (IDD). For example, caregiver-implemented AAC interventions, when supported by ABA principles, have been shown to enhance children’s ability to express needs and desires through alternative communication methods, which reduces maladaptive behaviors. It demonstrates that AAC, when integrated with functional communication training (a behavioral package rooted in ABA), can effectively replace problem behaviors with more functional communication strategies [57], joint attention, and social communication [58].

Traditional language interventions often focus independently on either verbal or imitative motor skills, but integrating ABA and AAC offers a multimodal approach that comprehensively enhances both communication and behavioral regulation, a strategy proven more effective in reducing problem behaviors among children with complex communication needs [57,59]. Beyond the positive results observed in this case study, the methods employed can be adapted for a range of populations, including children with more severe communication impairments or those with neurodevelopmental disorders such as autism spectrum disorder or intellectual disabilities [60]. Tailoring AAC strategies to the unique needs and abilities of each child is crucial. For example, children with severe impairments may benefit from high-tech AAC devices that provide more robust communicative options or from visual supports that improve comprehension [61]. Furthermore, interventions can incorporate multisensory approaches that engage both visual and auditory inputs, catering to diverse learning styles and communication needs [62]. The principles of ABA can also be applied flexibly to accommodate various levels of functioning, ensuring that all children have the opportunity to develop essential communication skills in a meaningful and effective way.

### 4.2. Effectiveness of AAC Techniques and Implications for Broader Applications

AAC includes all forms of communication that substitute oral language and/or writing to compensate for both temporary and permanent communication disabilities [27]. These techniques, particularly t personalized signs adapted to the child’s motor abilities, have been shown to promote functional communication and reducing problem behaviors, as confirmed by various studies [57,63]. Research suggests that customized AAC strategies, such as tailoring signs to each child’s motor abilities, increase the effectiveness of communication interventions and support sustainable behavioral improvements [30,64,65]. In this case, using marked syllables instead of phonemes facilitated a more natural verbal production process, making word articulation more accessible to the child. Sequential repetition of syllables helped improve memorization and automation of phono-articulatory patterns. Unlike phonemes, which are mental constructions, syllables correspond directly to individual articulatory actions, making them easier to identify and produce naturally for child [66].

Early intervention with AAC techniques can significantly improve both verbal and nonverbal communication skills in young children with communication difficulties. Light, et al. [67] introduced AAC methods to support early language development and reduce frustration-related behaviors. Studies further suggest that involving a child’s immediate social environment, including family members and educators, in AAC interventions promotes the generalization of communication skills, which is essential for maintaining progress outside the therapeutic setting [61,68]. This case study showed that the integration of ABA and AAC strategies, specifically marked syllables and personalized signs, improved functional communication and reduced problem behaviors in the child [67]. By associating visual and gestural cues with verbal expressions, C. expanded his repertoire of communication tools, shared by people in his daily life, such as family members and educators. This integration of visual and vocal elements aligns with findings from other studies, emphasizing the effectiveness of AAC in replacing maladaptive behaviors with functional communicative exchanges [57,63].

Additional research supports the use of gestural supports combined with verbal cues to promote communication in children with severe speech and language disorders. This approach is consistent with our use of signs appropriate to the child’s motor skills, reinforcing both motor imitation and communication skills within a comprehensive and individualized intervention plan [30]. Children with communication disabilities frequently display self- and hetero-aggressive behaviors as a means of seeking attention, gaining access to tangible objects, or avoiding undesirable situations [63]. However, these behaviors can be replaced with appropriate communicative exchanges, leading to a rapid decrease in problematic episodes. In this case study, the use of marked syllables increased all verbal operants and a reduced problem behaviors. After training, both elicited and spontaneous vocal productions increased, confirming the effectiveness of this approach [57]. The use of signs was directly related to the child’s emerging motor imitation skills, which already allowed for a form of communication based on combining gesture with vocalizing. Additionally, these signs, due to their visual and gestural nature, provided a shared means of communication within his social environment, including family members and educators [66].

At the conclusion of the 24-session treatment, significant improvements were observed in C.’s communicative abilities. His speech production increased both in isolation and in combination with signs used to express needs and desires. Moreover, the increase in vocal production remained steady and gradual beyond the end of the intervention. The VB-MAPP milestone score showed a notable improvement, indicating progress in both verbal and non-verbal communication skills, consistent with previous research on the positive impact of AAC on functional communication and the reduction of problem behaviors [57,59]. Additionally, there was a considerable decrease in the frequency of challenging behaviors, such as aggression and frustration, which are often associated with communication deficits in children with severe language impairments [63]. Although initially persistent, these behavior gradually decreased until they were no longer observed, which is a promising indicator of behavioral improvement. These findings are consistent with previous studies, which suggest that problem behaviors can be mitigated when functional communication skills are enhanced [59].

In conclusion, this case study confirms the effectiveness of an integrated approach using ABA and AAC strategies in improving communication and reducing challenging behaviors in children with SLD and GDD. By leveraging both motor and vocal elements, the intervention promoted a more functional and independent mode of communication for C., laying a foundation for further development. The combination of marked syllables and gestural support proved effective in bridging the gap between motor and verbal skills, offering a natural and accessible pathway to verbal communication for children with communication disabilities [66]. These results further support the growing body of literature on the benefits of AAC interventions in children with expressive language disorders [27,57,59].

## 5. Conclusions and Limitations

In this single-case study, the integration of ABA with AAC techniques, specifically marked syllables and signs, demonstrated significant improvements in both communicative abilities and behavioral regulation in a child diagnosed with SLD and GDD. The use of marked syllables provided a natural and accessible pathway to speech production, while the implementation of signs supported the child’s motor imitation skills, fostering functional communication. This combined approach not only led to an increase in verbal output but also contributed to a reduction in problem behaviors, such as frustration and aggression, commonly associated with communication deficits. These findings align with existing research and emphasize the importance of AAC strategies in promoting meaningful communication in children with severe language impairments. Despite these promising results, the single-case design of the study limits the generalizability of the findings, as outcomes may vary among individuals with similar diagnoses. Future research should consider larger sample sizes and control groups to better evaluate the effectiveness of the intervention in diverse populations. While the community context provides valuable insights into real-world applicability, it may introduce environmental variables that could influence outcomes. However, the success of this approach in a nonclinical setting suggests the potential for broader applications, including educational settings and home-based therapy.

Furthermore, this study highlights the potential for scaling these methods in community settings. Future interventions should consider the cost-effectiveness of implementing individualized AAC approaches, as they can significantly improve functional communication and reduce challenging behaviors while remaining accessible to a broader population. The insights gained from this case report could inform the development of AAC programs tailored to the specific needs of children with communication difficulties in community rehabilitation settings, ultimately enhancing the support available to families and practitioners.

Additionally, exploring the adaptability of augmentative gestural support across different age groups and neurodevelopmental conditions, such as autism spectrum disorder or intellectual disabilities, could further validate the effectiveness of these interventions and contribute to a more comprehensive understanding of their benefits in improving functional communication.

## Figures and Tables

**Table 1:** VB-MAPP explanatory table at baseline, after six months, and after twelve months.

	Time Points		
Skills	T0	T1	T2
Mand	No point on the scale	Emits at least 3 of these types of mands	Spontaneously emits 15 different mands
Tact	Tacts 4 reinforcing items without echoic prompts	Tacts 10 items without echoic prompts when tested	Tacts at least 200 nouns and/or verbs
Listener Responding	Attends to a speaker’s voice by orienting toward the speaker 5 times	Performs 4 different motor actions on command without a visual prompt	Selects items by color and shape from an array of 6 similar stimuli for 4 colors and 4 shapes
Visual Perceptual Skills And Matching-To-Sample (VP/MTS)	Visually tracks moving stimuli for 2 s, 5 times during the 30-min observation	Matches identical objects or pictures in a messy array of 6 for 25 items	Spontaneously matches any part of an arts and crafts activity to another person’s sample 2 times
Independent Play	Independently plays with 3 different items during a 30-min observation	Independently demonstrates the use of toys or objects according to their function for 5 items	Spontaneously engages in pretend or imaginary play on 2 occasions
Social Behavior and Social Play	Visually tracks and shows interest in people’s movement at least 2 times	Indicates that he wants to be held or physically played with 2 times	Spontaneously mands to peers 2 times
Motor Imitation	Imitates 2 gross motor movements when prompted with “Do this”.	Imitates 20 different fine motor actions	Spontaneously imitates 5 functional skills in the natural environment
Echoic	No point on the scale	Repeats vowels and syllables during evaluation	Repeat words with consonant groups and trisyllabies
Vocal	Spontaneously emits 10 different sounds with different intonation, with an average of 25 sounds in total per hour	Spontaneously gives 2 approximations of words	Spontaneously issues 15 different approximations of words identifiable during a 1-h observation
Listener Responding by Function, Feature, and Class (LRFFC)	Not assessed	Selects the correct item from an array of 8, for 25 different LRFFC fill-in statements of any type	Selects items from a book based on 2 verbal components: either a feature, function, or class for 25 LRFFC tasks
Intraverbal (IV)	Not assessed	Not assessed	Answers 25 different what questions.
Classroom Routines and Group Skills (Group)	Not assessed	Puts away personal items, lines up, or comes to a table, but it requires 2 or more prompts	Responds to 2 different group instructions or questions without direct prompts in a group of 3 or more children
Linguistic Structure	Not assessed	The child’s articulation of 5 tacts can be understood by familiar adults who cannot see the item tacted	Emits verb inflections for 10 past tense verbs, but not for 10 future tense verbs, or vice versa
Math	Not assessed	Identifies as a listener the numbers 1–5 in an array of 5 different numbers; numbering 1 to 5; counts from 1 to 5 objects, from a large set of objects with 1:1 match when requested verbally	Identifies as a listener 6 different comparisons involving measurement; denotes numbers 1 to 5

**Table 2:** Progression of the child’s behaviors and skills across different intervention time points (T0, T1, and T2).

	Time Points		
Barriers	T0	T1	T2
Behavior Problems	Behavioral problems many times a day	Frequent problem behaviors	Occasional short-lived problems with rapid return to typical condition
Instructional Control	Wide range of situations that evoke non-cooperation and problem behaviors issued to avoid or run away from the request	Wide range of situations that evoke non-cooperation and problem behaviors issued to avoid or run away from the request	Some requests evoke non-cooperation but usually collaborate with instructions
Defective Mand	It does not have functional mand	It does not have functional mand	Produces mand but has higher scores in listener skills
Defective Tact	Tact clearly deficient in generalization, spontaneity, and avoidance during training	Tact clearly deficient in generalization, spontaneity, and avoidance during training	Can name objects but listener’s abilities are superior
Defective Echoic	Failure in echoic abilities despite good imitative abilities	Echoic repertory limited	Repeats correctly, but the repertoire is lower than other skills
Defective Imitation	Imitates but has difficulty in fine-motor imitations	Imitates but has difficulty in fine-motor imitations	Evolving imitation abilities
Defective VP-MTS	Ability to match sample stimuli, but avoidance during training	Ability to match sample stimuli, but avoidance during training	Show skill when matching
Defective Listener	Listener discrimination ability but negative behaviors	Listener discrimination ability, but negative behaviors	Discrimination skills, but lower scores than other milestrone
Defective Intraverbal	Fails to acquire intraverbal behaviors	Fails to acquire intraverbal behaviors	Shows intraverbal responses, but the score is lower than other milestones
Defective Social Skills	Rarely starts social interactions	Rarely starts social interactions	Constantly growing social skills

**Table 3:** Griffiths Mental Developmental Scale-III at T1 and T2. GMDS-III was used as a tool for assessing the child’s development. The child completed the items placed in an ascending difficulty order. For each subscale, the child reached a ceiling representing that skill’s developmental quotient.

GDMS III Scale	T1	T2
General Quotient	24	43
Foundations of Learning	25	48
Language and Communication	20	42
Eye and Hand Coordination	25	48
Personal–Social–Emotional	18	44
Gross Motor	31	37

## Data Availability

Data are available upon request.
